# Divergent Expression Patterns and Function of Two *cxcr4* Paralogs in Hermaphroditic *Epinephelus coioides*

**DOI:** 10.3390/ijms19102943

**Published:** 2018-09-27

**Authors:** Wei-Jia Lu, Li Zhou, Fan-Xiang Gao, Zhi-Hui Sun, Zhi Li, Xiao-Chun Liu, Shui-Sheng Li, Yang Wang, Jian-Fang Gui

**Affiliations:** 1 State Key Laboratory of Freshwater Ecology and Biotechnology, Institute of Hydrobiology, Chinese Academy of Sciences, Wuhan 430072, China; a18914926176@163.com (W.-J.L.); zhouli@ihb.ac.cn (L.Z.); gaofanxiang@hhu.edu.cn (F.-X.G.); lizhi@ihb.ac.cn (Z.L.); 2 University of Chinese Academy of Sciences, Beijing 100049, China; 3 Key Laboratory of Mariculture & Stock Enhancement in North China Sea, Ministry of Agriculture and Rural Affairs, Dalian Ocean University, Dalian 116023, China; huihappy1012@126.com; 4 State Key Laboratory of Biocontrol, Guangdong Province Key Laboratory for Aquatic Economic Animals, and Institute of Aquatic Economic Animals, School of Life Sciences, Sun Yat-Sen University, Guangzhou 510275, China; lsslxc@mail.sysu.edu.cn (X.-C.L.); lishuisheng219@126.com (S.-S.L.)

**Keywords:** *cxcr4*, paralog, biased and divergent expression, chemotaxis ability, orange-spotted grouper, sub-functionalization

## Abstract

Chemokine receptor Cxcr4 evolved two paralogs in the teleost lineage. However, *cxcr4a* and *cxcr4b* have been characterized only in a few species. In this study, we identified two *cxcr4* paralogs from the orange-spotted grouper, *Epinephelus coioides*. The phylogenetic relationship and gene structure and synteny suggest that the duplicated *cxcr4a/b* should result from the teleost-specific genome duplication (Ts3R). The teleost *cxcr4* gene clusters in two paralogous chromosomes exhibit a complementary gene loss/retention pattern. *Ec_cxcr4a* and *Ec_cxcr4b* show differential and biased expression patterns in grouper adult tissue, gonads, and embryos at different stages. During embryogenesis, *Ec_cxcr4a/b* are abundantly transcribed from the neurula stage and mainly expressed in the neural plate and sensory organs, indicating their roles in neurogenesis. *Ec_*Cxcr4a and *Ec_*Cxcr4b possess different chemotactic migratory abilities from the human SDF-1α, *Ec_*Cxcl12a, and *Ec_*Cxcl12b. Moreover, we uncovered the N-terminus and TM5 domain as the key elements for specific ligand–receptor recognition of *Ec_*Cxcr4a-*Ec_*Cxcl12b and *Ec_*Cxcr4b-*Ec_*Cxcl12a. Based on the biased and divergent expression patterns of *Eccxcr4a*/*b*, and specific ligand–receptor recognition of *Ec_*Cxcl12a/b–*Ec_*Cxcr4b/a, the current study provides a paradigm of sub-functionalization of two teleost paralogs after Ts3R.

## 1. Introduction

Chemokine receptors belong to the largest rhodopsin family of G-protein coupled receptors (GPCR) [1,2] and are considered an evolutionary innovations in vertebrate [3]. As key regulators of cell migration, they interact with ligand chemokines and exert pivotal roles in diverse physiological processes, including inflammatory and immune responses, angiogenesis and hematopoiesis, neurological development and neuroendocrine regulation, organogenesis, and germ cell migration [4,5,6,7,8,9]. Depending on the chemokine subfamily they bind, chemokine receptors are classified into the CXCR, CCR, CX3CR and XCR subfamilies [9]. Accompanied by the emergence of thymopoiesis in jawed vertebrates, CXCL12-CXCR4 is one of the most ancient chemokine ligand–receptor in vertebrates [3,10,11] and it participates in angiogenesis, lymphopoiesis, neurogenesis, myelopoiesis, germ cell development, and so on [12,13,14,15,16,17,18].

In the teleost lineage, both *cxcr4* and *cxcl12* have evolved two paralogs [19,20,21,22] in the course of fish-specific whole genome duplication [23,24]. *Cxcr4a* and *cxcr4b* have been identified in zebrafish (*Danio rerio*) [25,26], Asian swamp eel (*Monopterus albus*) [27], and channel catfish (*Ictalurus punctatus*) [28]. In addition, one paralog of *cxcr4* has been characterized in several telesots [29,30,31,32,33,34,35,36]: medaka (*Oryzias latipes*) [29], turbot (*Scophthalmus maximus*) [30], orange-spotted grouper (*Epinephelus coioides*) [31], and so on. The expression and function of *cxcl12s*-*cxcr4s* have been well documented in zebrafish [4]. Additional studies have focused on the dynamic expression changes in response to pathogens or hypoxia stress [28,30,31,32,33,34,35,36,37,38]. However, detailed research on comparative synteny and evolution analysis, and divergent expression patterns and function of the two *cxcr4* paralogs in teleost is scarce.

Orange-spotted grouper is a protogynous hermaphrodite teleost with high commercial value, widely cultured in Southeast Asia [39,40,41,42,43,44]. The repertoire of chemokine receptors varies in each species owing to their rapid evolution through species-specific gene duplications [3]. In a previous study, one paralog of *cxcr4*, gCXCR4, was identified in orange-spotted grouper and revealed its up-regulated expression in response to lipopolysaccharide (LPS) and nervous necrosis virus (NNV) infection [31]. However, whether another paralog of *cxcr4* exists in the grouper genome, and the divergent expression pattern and function between the two paralogs in grouper are unclear. In this study, we characterized two paralogs of *cxcr4* in orange-spotted grouper, and revealed their expression in grouper adult tissue, embryos, and gonads at different developmental stages. Moreover, we identified two paralogs of *cxcl12* and revealed the key elements for specific ligand–receptor recognition of grouper *cxcr4a/b-cxcl12a/b*.

## 2. Results

### 2.1. Molecular Characterization of Two cxcr4 Paralogs in Orange-Spotted Grouper

**Figure 1:**
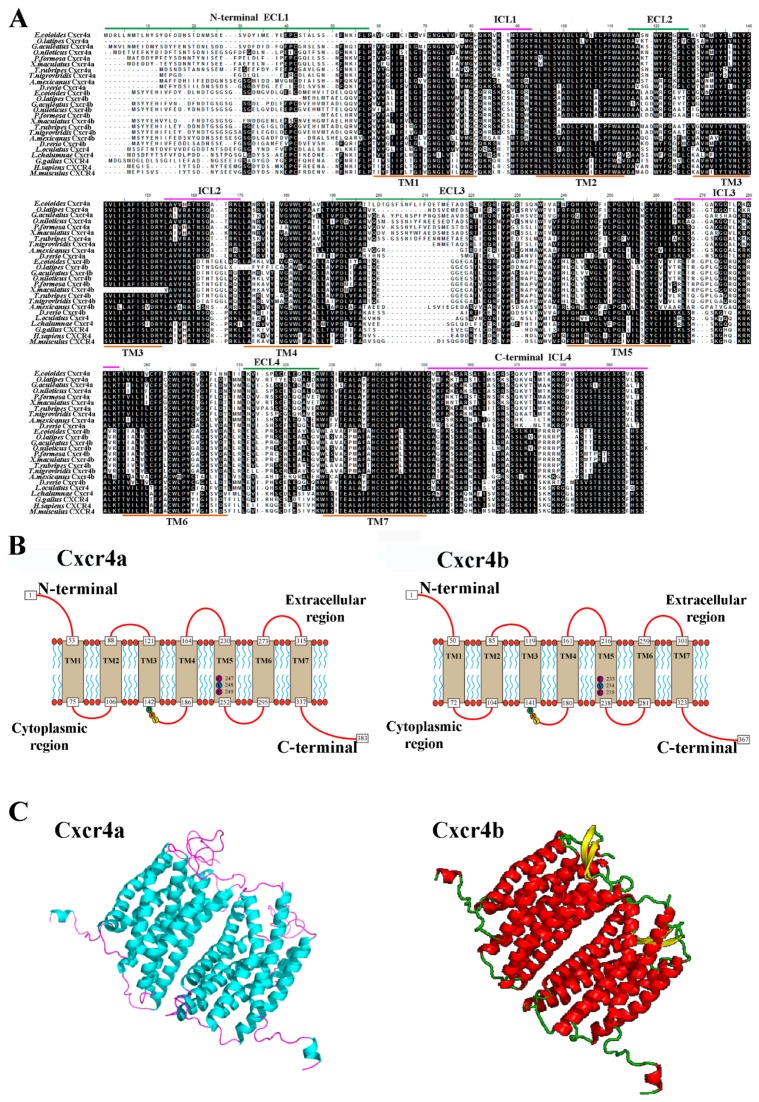
Molecular characterization of orange-spotted grouper Cxcr4a and Cxcr4b. (**A**) Multiple amino acid sequence alignment of *Ec_*Cxcr4a/b protein and other vertebrate Cxcr4 proteins. TM, Transmembrane domains; ECL, extracellular loop; ICL, intracellular loop. (**B**) Diagrammatic representation of the domain structure and the topology of transmembrane regions of *Ec_*Cxcr4a/b. TM helices are shown as light brown barrels, and ECLs and ICLs are shown as red lines. Specific conserved motifs, such as DRY and CYC, are also indicated. (**C**) The 3-D structures of *Ec*Cxcr4a/b. Alpha helices are shown as blue or red helices and beta sheets are shown as yellow arrows.

Two *cxcr4* genes (EPI_005803 and EPI_021273) were cloned from orange-spotted grouper. The full-length cDNA of EPI_005803 is 1794 bp, consisting of a 116-bp 5’-terminal untranslated region (UTR), a 1152-bp open-reading frame (ORF), and a 526-bp 3’-UTR (Figure S1). The EPI_021273 cDNA is 1293 bp in length, and encodes 367 amino acids (Figure S2). To clarify the structural features of orange-spotted grouper *cxcr4a/b*, 23 *cxcr4* genes from 14 species were downloaded from the Ensembl and NCBI databases, and we performed multiple sequence alignments with *Ec_cxcr4a* and *Ec_cxcr4b* (Figure 1A). Tetrapods, coelacanth (*Latimeria chalumnae*) and spotted gar (*Lepisosteus oculatus*) possess only one *cxcr4* gene, while other teleosts have two *cxcr4* genes. EPI_005803 and EPI_021273 share about 60% and 50% identities with homologs of tetrapod, coelacanth and spotted gar, respectively, so we named them as *Ec_cxcr4a* (EPI_005803) and *Ec_cxcr4b* (EPI_021273). The identity between *Ec_*Cxcr4a and *Ec_*cxcr4b is 51.1%. Both *Ec_*Cxcr4a and *Ec_*Cxcr4b consist of seven hydrophobic transmembrane (TM) domains, four extracellular loops (ECL) and four intracellular loops (ICL), and an extracellular N-terminus and an intracellular C-terminus (Figure 1A,B). By using the SWISS-MODEL, 3D protein-structure models of *Ec_*Cxcr4a and *Ec_*Cxcr4b were predicted (Figure 1C). Both *Ec_*Cxcr4a and *Ec_*Cxcr4b are homodimers and contain seven α-helices in each homolog that correspond to seven TM domains. Additionally, *Ec_*Cxcr4b possesses two β-sheets in each homolog.

### 2.2. Phylogenetic Relationship, Gene Structure and Gene Synteny of Teleost and Tetrapod cxcr4s

**Figure 2:**
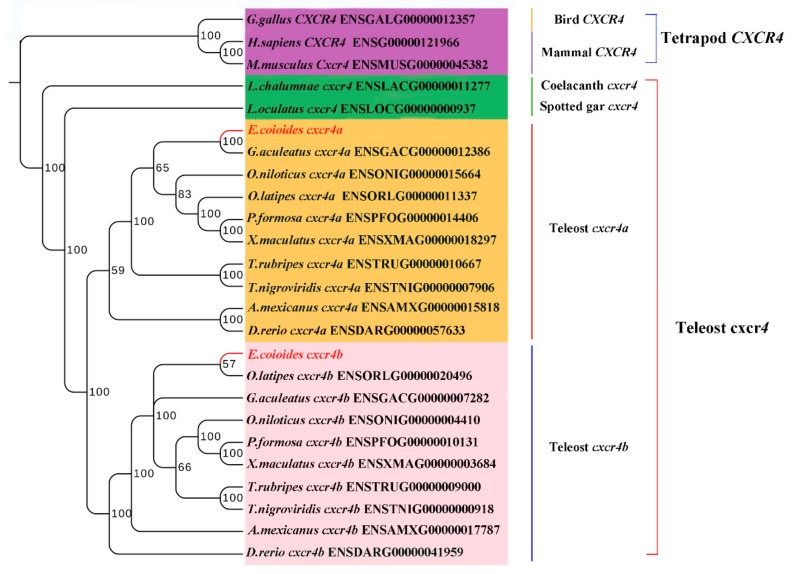
Phylogenetic tree of vertebrate Cxcr4. Phylogenetic analysis showed that teleost Cxcr4 separated into two groups (Cxcr4a and Cxcr4b). Bayesian posterior probability values are indicated.

To confirm the nomenclature and resolve the origin of the duplicated *Ec_cxcr4a/b*, a phylogenetic tree was constructed (Figure 2). Vertebrate Cxcr4s clustered largely consistent with the accepted species phylogeny and were divided into two clearly distinct clusters of the teleost and tetrapod Cxcr4. *Ec_*Cxcr4a and *Ec_*Cxcr4b are clustered into teleost Cxcr4a and teleost Cxcr4b branch, respectively. Teleost Cxcr4a and Cxcr4b branches are first grouped together and clustered with coelacanth and spotted gar Cxcr4, and then clustered with the tetrapod Cxcr4 branch.

**Figure 3:**
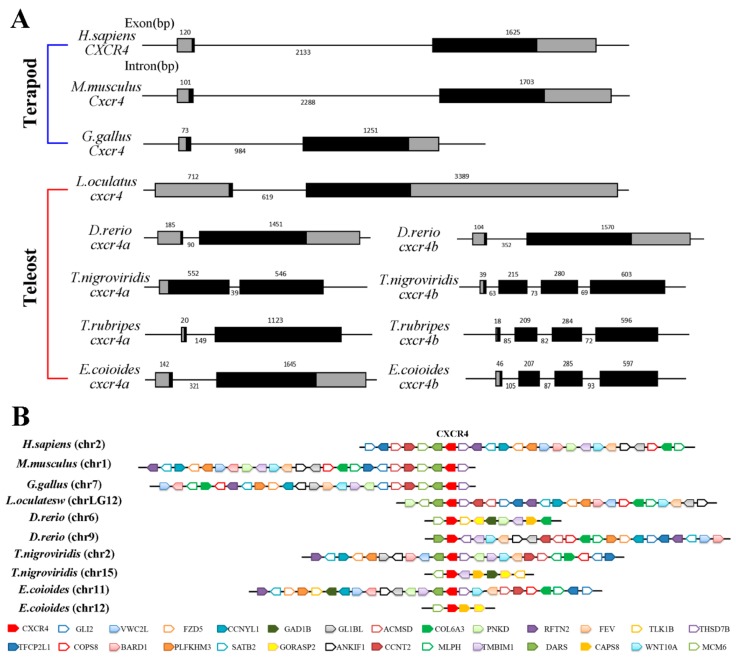
Genomic structure and gene synteny of vertebrate *cxcr4*. (**A**) Genomic structure of *cxcr4*. Exons and introns are shown by boxes and horizontal lines, respectively. Open-reading frames (ORFs) are highlighted by black boxes. Exon and intron size are indicated above or below as bp. (**B**) Syntenic alignment of chromosomal regions around vertebrate *cxcr4* genes. Chromosome segments are represented as thick lines. Conserved gene blocks are shown in matching colors and transcription orientations are indicated by arrows. Chr, chromosome.

Subsequently, the genomic sequences of around *cxcr4* genes from seven species were downloaded and their gene structure and syntenic alignment were analyzed (Figure 3). *Ec_cxcr4a* has a bi-exonic structure and its ORF is interrupted by an intron, same as tetrapod *cxcr4s* [34], spotted gar *cxcr4*, and other teleost *cxcr4as*. *Ec_cxcr4b* is composed of four exons and three introns, consistent with other teleost *cxcr4bs* except zebrafish *cxcr4b* (Figure 3A). *Ec_cxcr4a* and *Ec_cxcr4b* are located on chromosome 11 and 12 of the orange-spotted grouper, respectively. Although identical genes were found in human (*Homo sapiens*) chromosome 2 (chr2), mouse (*Mus musculus*) chr1, chicken (*Gallus gallus*) chr7, and spotted gar chrLG12, the different locations of identical genes among these chromosomes showed that a large number of rearrangements occurred during evolution. However, a block of a gene cluster (*MCM6*-*DARS*-*CXCR4*-*THSD7β*) around *cxcr4* loci presents conserved synteny in tetrapods and spotted gar (Figure 3B). Compared with the genes in tetrapod and spotted gar chromosomes, a complementary loss/retention pattern of genes exists between two teleost paralogous chromosomes. Conserved gene clusters (*dars*-*cxcr4a*-*thsd7β*) and (*mcm6*-*cxcr4b*) are found in two paralogous Tetraodon and orange-spotted grouper chromosomes, respectively (Figure 3B). Compared to the genes in spotted gar chrLG12, not only a large number of rearrangements, but also many gene inversions occurred in orange-spotted grouper chr11 and chr12.

### 2.3. Biased and Differential Expression Patterns of Two cxcr4 Paralogs in Orange-Spotted Grouper

**Figure 4:**
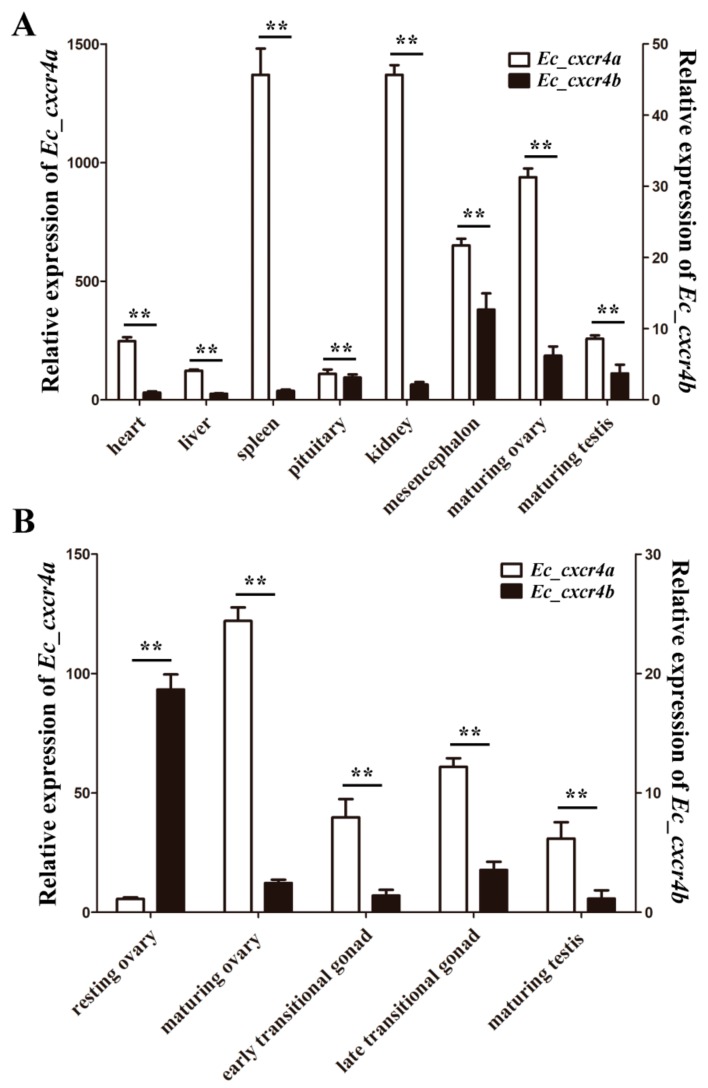
*Ec_cxcr4a* and *Ec_cxcr4b* expression: in adult tissue (**A**); and gonads at different developmental stages (**B**). β-actin was used as control. Each bar represents mean ± standard deviation (SD) (n = 3). Asterisks (**) indicate the significant differences between *Ec_cxcr4a* and *Ec_cxcr4b* (*p* < 0.01). Data were acquired from three independent experiments.

The distributions of *Ec_cxcr4a* and *Ec_cxcr4b* in adult orange-spotted grouper tissue were analyzed by quantitative RT-PCR (qPCR). *Ec_cxcr4a* and *Ec_cxcr4b* were detected in all eight examined tissues. The expression levels of *Eccxcr4a* in adult tissue are all remarkably higher (51–1093 folds) compared to *Ec_cxcr4b* (*p* < 0.01), indicating significantly biased expression of *Ec_cxcr4a* and *Ec_cxcr4b* in adult tissue. In addition, *Ec_cxcr4b* is most abundantly expressed in the mesencephalon, while *Ec_cxcr4a* is highly expressed in the spleen and kidney, suggesting *Ec_cxcr4a* might play an important role in immune response (Figure 4A).

Owing to differential expression of *Eccxcr4a/b* in maturing ovary and testis (Figure 4A), we also evaluated the expression of *Ec_cxcr4a* and *Ec_cxcr4b* in the gonads at five developmental stages (Figure 4B). A resting ovary contains a large number of primary-growth oocytes (PO) and previtellogenic oocytes (PVO). In maturing ovaries, many vitellogenic oocytes (VO), PVO, and PO fill the gonad. The degenerating primary oocytes (DPO), PO, spermatocytes (SPC), and spermatids (SPD) are mixed in lobules of early transitional gonads. Along with sex transition, many spermatogonia (SPG), SPC, and SPD arise, and only a few PO scatter in the lobules of late transitional gonads. In mature testis, male germ cells at all spermatogenic stages scatter within the cysts [45,46]. *Ec_cxcr4b* transcripts are highly expressed in the resting ovary, and sharply decrease by 86.8% in the maturing ovary. However, *Ec_cxcr4a* exhibit a distinctly differential expression pattern from *Ec_cxcr4b*. *Ec_cxcr4a* expression is relatively lower in the resting ovary, and sharply increase up to 22-folds in the maturing ovary. When the ovary begins to cause sex transition to the testis, *Ec_cxcr4a* expression decrease but still keeps a relative higher level (17–28 folds) in transitional gonads and maturing testis compared to those of *Eccxcr4b* (*p* < 0.01).

**Figure 5:**
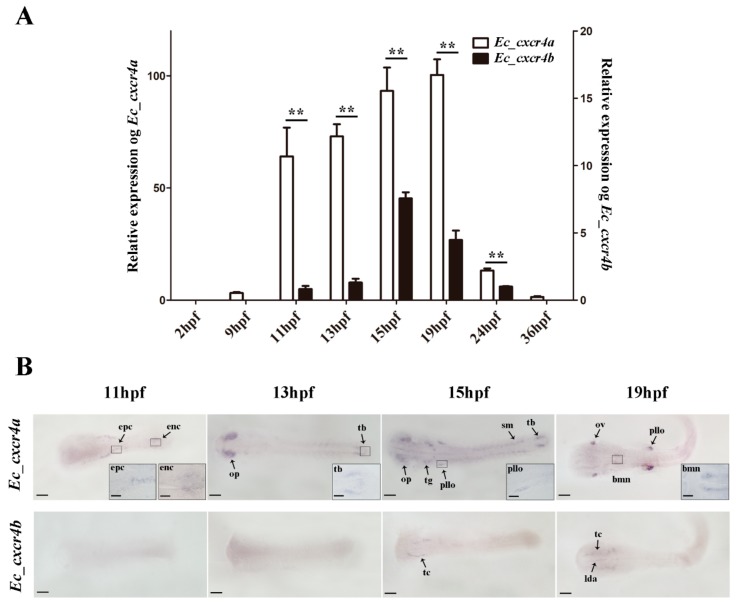
*Ec_cxcr4a* and *Ec_cxcr4b* expression during embryogenesis. (**A**) Quantitative RT-PCR (qPCR) detection of *Ec_cxcr4a* and *Ec_cxcr4b* transcripts from 2 hpf to 36 hpf. EF1α was used as control. Each bar represents mean ± SD (n = 3). The asterisks (**) indicate the significant differences between *Ec_cxcr4a* and *Ec_cxcr4b* (*p* < 0.01). (**B**) Whole-mount in situ hybridization (WISH) detection of *Ec_cxcr4a* and *Ec_cxcr4b* in orange-spotted grouper embryos. Development stages are marked on top of panels. Enc, endothelial cell; epc, epithelial cell; op, olfactory placode; tb, tail bud; pllo, primordium lateral line organ; tg, trigeminal ganglion; sm, somites; bmn, brachiomotor neurons; ov, otic vesicle; tc, telencephalon; lda, lateral dorsal aorta. Bar: 50 μm; magnification bar: 10 μm.

The dynamic expression changes of *Ec_cxcr4a* and *Ec_cxcr4b* during embryogenesis were analyzed by qPCR and whole-mount in situ hybridization (WISH) (Figure 5). *Ec_cxcr4a* was transcribed at 9 h post fertilization (hpf), sharply increasing expression up to 20-fold at 11 hpf, and then kept a high level until 24 hpf. *Ec_cxcr4b* expression was hardly detected until 11 hpf, gradually increased, reached a peak at 15 hpf, and then gradually decreased during 19–24 hpf. *Ec_cxcr4a* and *Ec_cxcr4b* also showed an apparently biased expression pattern during embryogenesis (Figure 5A). *Ec_cxcr4a* transcripts were localized in epithelial cells and endothelial cells at 11 hpf and were predominantly expressed in the olfactory placode at 13 hpf. In addition, positive signals were also observed in tail buds). At 15 hpf, *Ec_cxcr4a* transcripts were located in the primordium lateral line organ, trigeminal ganglion and somites in addition to the olfactory placode and tail bud. Along with the development progress of embryos, positive signals were concentrated in the otic vesicle, brachiomotor neurons and primordium lateral line organ (Figure 5B). Faint *Ec_cxcr4b* signals were observed in the neural plate at 13 hpf and concentrated in the telencephalon at 15 hpf. In addition, positive signals emerged in the telencephalon and lateral dorsal aorta at 19 hpf.

### 2.4. Differential Chemotactic Migratory Abilities of HEK293T Cells Transfected with Cxcr4a/b Induced by Cxcl12a/b

**Figure 6:**
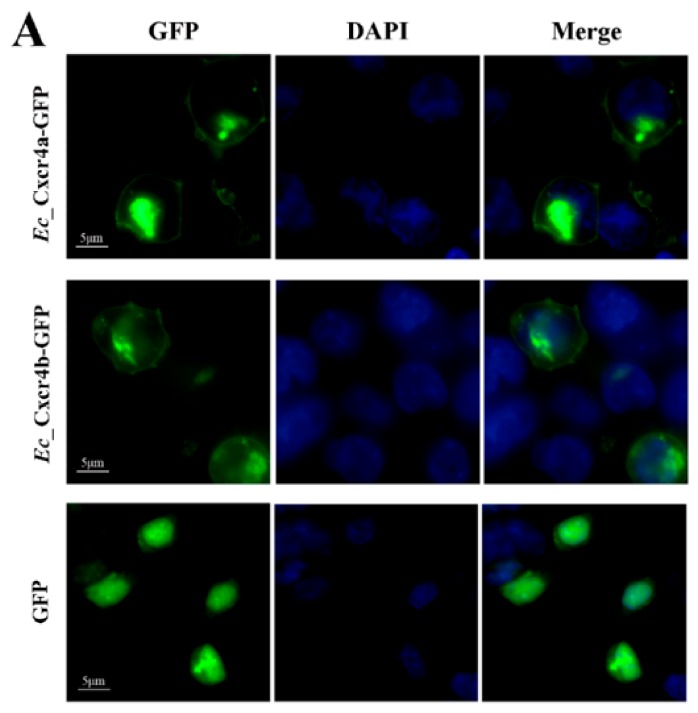

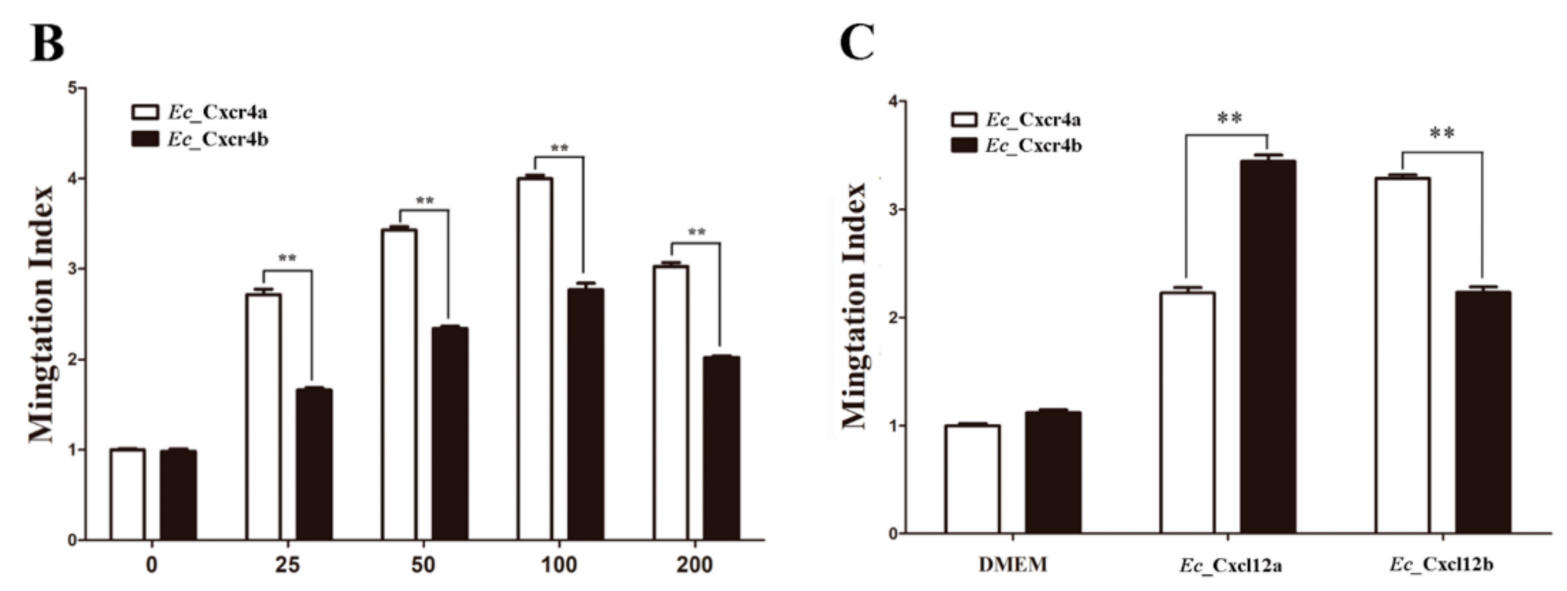
Subcellular localization and chemotaxis ability of *Ec_cxcr4a* and *Ec_cxcr4b*. (**A**) *Ec_*Cxcr4a and *Ec_*Cxcr4b localization in HEK293T cells. Bar = 5 μm. (**B**,**C**) The chemotactic migration of *Ec_cxcr4a*-HEK293T and *Ec_cxcr4b*-HEK293T toward human SDF-1 (**B**), or *Ec_*Cxcl12a-HEK293T or *Ec_*Cxcl12ab-HEK293T (**C**) Each bar represents mean ± SD (n = 3). Asterisks (**) indicate the significant differences between *Ec_cxcr4a* and *Ec_cxcr4b* (*p* < 0.01). DMEM, Dulbecco’s Modified Eagle Medium. Serum-free DMEM was placed in the lower chamber as a control.

Cells that strongly express human CXCR4 can migrate towards CXCL12 (also known as SDF-1) in vitro [47]. To evaluate the difference of chemotactic migratory abilities between *Ec_cxcr4a* and *Ec_cxcr4b*, wild-type *Ec_cxcr4a* or *Ec_cxcr4b* (WTs) were linked to GFP (*Ec_cxcr4a*-GFP and *Ec_cxcr4b*-GFP) and transfected into HEK293T cells, respectively. Both *Ec_*Cxcr4a-GFP and *Ec_*Cxcr4b-GFP mainly localized in the cell membrane and some region of the internal membrane system (Figure 6A). A transwell migration assay was used to quantitatively evaluate the chemotactic migration ability of HEK293T cells overexpressing *Ec_*Cxcr4a-GFP (*Ec_*Cxcr4a-HEK293T) or *Ec_*Cxcr4b-GFP (*Ec_*Cxcr4b-HEK293T) in response to human SDF-1α in vitro. In comparison with serum-free DMEM, both of the average percentages of *Ec_*Cxcr4a-HEK293T and *Ec_*Cxcr4b-HEK293T that migrated toward human SDF-1α significantly increased in a dose-dependent manner (Figure 6B), indicating the chemotaxis of SDF-1 on *Ec_*Cxcr4a and *Ec_*Cxcr4b. The overexpression of *Ec_*Cxcr4a could make remarkably more HEK293T cells (1.4–1.6 fold) migrate to human SDF-1α than that induced by the overexpression of *Ec_*Cxcr4b (*p* < 0.01).

To test the differential chemotactic migratory abilities between *Ec*Cxcr4a and *Ec*Cxcr4b in response to their ligands, *cxcl12* cDNAs were cloned from orange-spotted grouper by RACE PCR based on the conserved sequences among teleost *cxcl12s*. Two divergent cDNAs of *cxcl12* were also identified (Figures S3–S6). Subsequently, *Ec_*Cxcl12a-HEK293T or *Ec_*Cxcl12b-HEK293T was placed in the lower chamber, and *Ec_*Cxcr4a-HEK293T and *Ec_*Cxcr4b-HEK293T were placed in the upper chamber. The migration ability of *Ec_*Cxcr4a-HEK293T toward *Ec_*Cxcl12b-HEK293T increased to 3.3-fold after 24 cultured hours, while that of *Ec_*Cxcr4a-HEK293T toward *Ec_*Cxcl12a-HEK293T rose up 2.2-fold (Figure 6C), indicating stronger chemotaxis of *Ec_*Cxcl12b on *Ec_*Cxcr4a (*p* < 0.01). Similarly, *Ec_*Cxcr4b showed higher chemotactic response to *Ec_*Cxcl12a.

### 2.5. Specific Key Domains for Chemotactic Interaction of Ec_Cxcr4a/b-Ec_Cxcl12a/b

**Figure 7:**
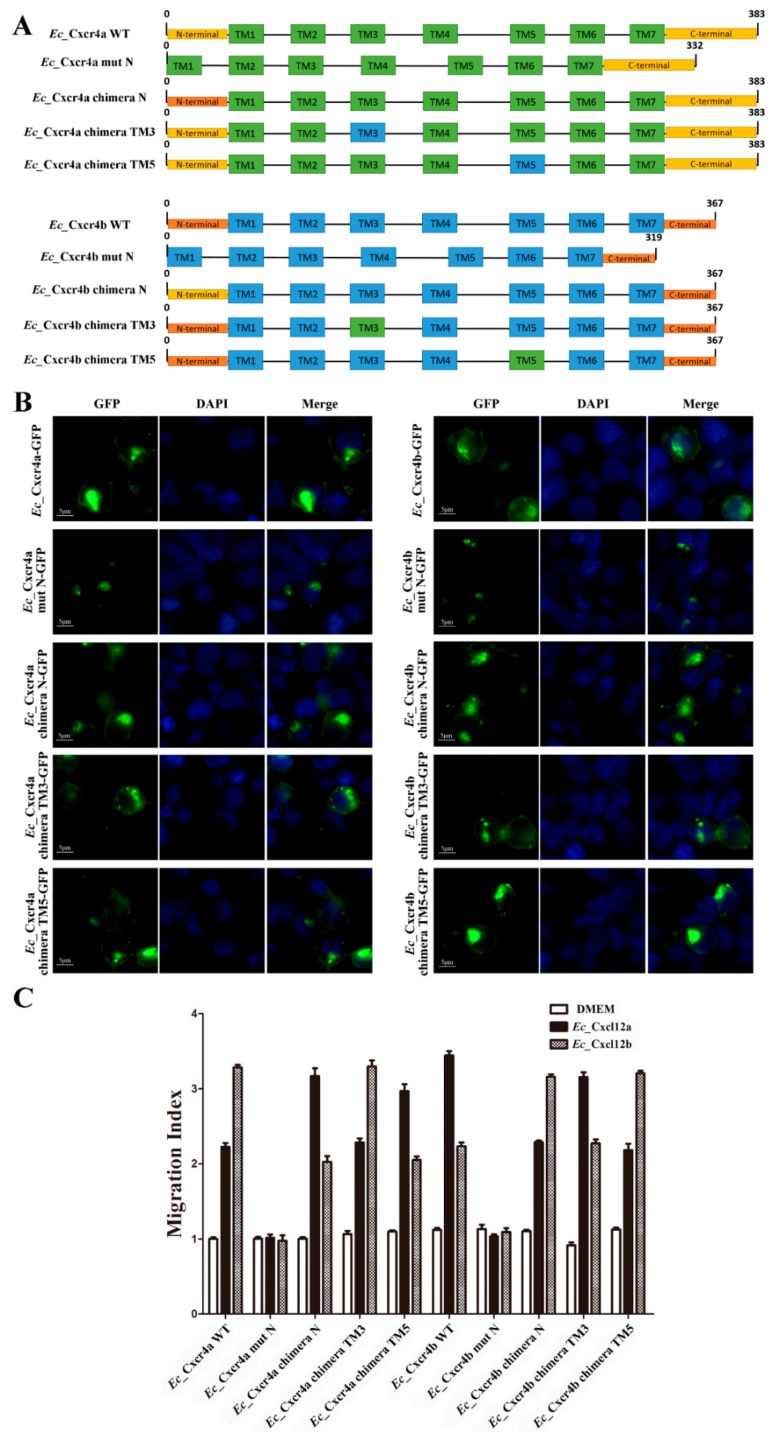
Subcellular localization and chemotaxis ability of *Ec_*Cxcr4a/b chimeras and mutants. (**A**) Schematic diagrams of wild-type (WT) and mutated *Ec_*Cxcr4a and *Ec_*Cxcr4b structures. Transmembrane domains (TMs) are illustrated by green or blue boxes, and the N-terminal and C-terminal are represented by yellow or orange boxes, respectively. Numbers refer to amino acid residues. (**B**) Localization of mutated *Ec_*Cxcr4a and *Ec_*Cxcr4b in HEK293T cells. Bar = 5 μm. (**C**) Chemotactic migration of HEK293T cells over-expressing mutated *Ec_*Cxcr4a or *Ec_*Cxcr4b toward *Ec_*Cxcl12a-HEK293T or *Ec_*Cxcl12ab-HEK293T. Serum-free DMEM was placed in the lower chamber as a control.

In mammals, the extracellular N-terminus, ICL2, and ECL3 have shown to be important to the CXCR4-CXCL12 interaction and activation of downstream pathways [48,49]. To reveal the specific key domains for the chemotactic interaction of *Ec_*Cxcr4b-*Ec_*Cxcl12a and *Ec_*Cxcr4a-*Ec_*Cxcl12b, respectively, we generated eight mutants by truncating the N-terminus of *Ec_*Cxcr4a and *Ec_*Cxcr4b, or by replacing the corresponding N-terminus, TM3 and TM5 of *Ec_*Cxcr4a and *Ec_*Cxcr4b (Figure 7A). Both *Ec_*Cxcr4a mut N-GFP and *Ec_*Cxcr4b mut N-GFP were mainly distributed in cytoplasm, not in cell membrane. The GFP signals of the six other mutants were still localized to the cell membrane and inner membrane, similar to WT *Ec_*Cxcr4a-GFP and *Ec_*Cxcr4b-GFP (Figure 7B). The results indicate that the N-terminus is crucial for the membrane localization of *Ec_*Cxcr4a/b.

Then, we assessed the chemotaxis abilities of these mutants in response to their ligand chemokines, *Ec_*Cxcl12a or *Ec_*Cxcl12b, in comparison with WTs. *Ec_*Cxcl12a-HEK293T or *Ec_*Cxcl12b-HEK293T were placed in the lower chamber and HEK293T cells expressing mutants were placed in the upper chamber. After 24 cultured hours, *Ec_*Cxcr4a mut N-GFP and *Ec_*Cxcr4b mut N-GFP lost their chemotaxis abilities. The chemotaxis abilities of the *Ec_*Cxcr4a chimera TM3-GFP and *Ec_*Cxcr4b chimera TM3-GFP to *Ec_*Cxcl12a-GFP and *Ec_*Cxcl12b-GFP were identical to the WTs, suggesting that the replacement of the TM3 domain did not influence the specific chemotactic interactions of *Ec_*Cxcr4b-*Ec_*Cxcl12a and *Ec_*Cxcr4a-*Ec_*Cxcl12b. In contrast, HEK293T cells expressing the *Ec_*Cxcr4a chimera N-GFP showed higher chemotaxis to *Ec_*Cxcl12a-GFP (3.2 fold) than that of *Ec_*Cxcr4a-GFP to *Ec_*Cxcl12a-GFP (2.0 fold), and lower chemotaxis to *Ec_*Cxcl12b-GFP (2.2 fold) than that of *Ec_*Cxcr4a-GFP to *Ec_*Cxcl12b-GFP (3.3 fold) (Figure 7C). The switch of chemotaxis effects was also observed in the *Ec_*Cxcr4b chimera N-GFP, *Ec_*Cxcr4a chimera TM5, and *Ec_*Cxcr4b chimera TM5.

## 3. Discussion

In addition to the two rounds of whole genome duplication (WGD) that occurred at the root of vertebrate lineage, the fish-specific genome duplication that occurred in a teleost ancestor gave dramatic rise and rapid radiation to teleost fish [50,51,52,53,54,55]. The phylogenetic tree showed that teleost Cxcr4 separated into two groups (Cxcr4a and Cxcr4b). As with tetrapod, coelacanth and spotted gar diverged from teleosts before Ts3R [51,56], and have only a single *cxcr4* gene that falls outside the branches of teleost *cxcr4s* (Figure 2). The results suggest that the duplicated *cxcr4a/b* should have been produced through Ts3R, which is also supported by the duplication of neighboring genes surrounding *cxcr4a/b* (Figure 3B). Together with highly conserved gene synteny among homologous chromosomes containing teleost *cxcr4a* and tetrapod *cxcr4* (Figure 3B), we supposed that the tetrapod *cxcr4* cannot come from only one of the two teleost duplicated genes. The rapid rediploidization process was demonstrated to cause complicated and non-Mendelian genomic changes just following WGD events, including duplicated gene loss, additional gene duplication, gene inversion and large genomic reorganization [57,58,59,60,61,62]. The gene is ancestral. One of the duplicated genes kept more similar features with one of the ohnologous copies [62,63]. Through gene synteny analysis, we not only revealed a complementary gene loss/retention pattern, but also observed many gene inversions, genomic rearrangements and gene translocations in two teleost paralogous chromosomes (Figure 3B), indicating divergent evolution occurring in the radiation of teleost.

During rediploidization, pseudogenized genes lost function, or underwent sub-functionalization to partition ancestral gene function, or neo-functionalization to obtain a new function [57,58,60,63]. Two paralogs generally showed a dominant, biased or silencing expression pattern [64,65,66,67,68,69,70,71,72,73,74,75,76]. In this study, we revealed a significantly biased expression of *Eccxcr4a* and *Ec_cxcr4b* in adult tissue, gonads and embryos at different stages. The biased expression of two *cxcr4* paralogs had been observed in zebrafish embryogenesis [25] and several Asian swamp eel adult tissue [27]. Teleost *cxcr4s* are constitutively expressed in adult tissue, and highly constitutive and up-regulated in immune tissue after pathogen infection [27,30,32,33,34,36,38,77,78], which indicates their role in immune surveillance and response. *Ec_cxcr4a* is highly expressed in analyzed tissues, while *Ec_cxcr4b* is mainly expressed in the mesencephalon (Figure 4A). Thus, it seems that *Ec_cxcr4a* acts the main role to execute Cxcr4 functions in orange-spotted grouper. Interestingly, we observed distinctly differential expression patterns between *Ec_cxcr4a* and *Ec_cxcr4b* during sex differentiation and sex reversal, indicating *Ec_cxcr4a/b* might play a role in gonad differentiation (Figure 4B). Cxcr4 has been proven to guide the migration of primordial germ cells (PGCs) [26,77,79,80,81]. However, the function of *cxcr4* in gonad differentiation is still mostly unknown, owing to the severe defects and perinatal death of *Cxcr4* knockout mice [82,83]. The function of *Ec_cxcr4a/b* in grouper sex differentiation awaits further investigation.

Previous studies have shown that zebrafish *cxcr4s* are essential for normal development, including the migration of PGCs [17,26,29] and endodermal cells [84], muscle formation [85], and sensory development and retinal growth [86,87]. The expression pattern of *cxcr4a/b* during embryogenesis has been finely described only in zebrafish [25]. Different from zebrafish, the transcripts of *Ec_cxcr4a* and *Ec_cxcr4b* were detected until the gastrula stage (9 hpf) and neurula stage (11 hpf), respectively (Figure 5A). It was supposed that two zebrafish paralogs partitioned ancestral functions, of which *cxcr4a* maintained most of murine Cxcr4 functions [88], while *cxcr4b* acquired a novel early function [25]. However, the distribution of *Ec_cxcr4a* in grouper embryos was more reminiscent of zebrafish *cxcr4b*. They are both expressed in structures associated with sensory organs or escape reactions (Figure 5B). In addition, *Ec_cxcr4b* and zebrafish *cxcr4b* were co-localized in the telencephalon. CXCL12-CXCR4 signaling is required for neuronal specification, migration, and outgrowth [88,89,90,91,92]. Therefore, the abundant expression of *Ec_cxcr4a/b* in the neural plate and sensory organs suggests that they might play a role in grouper neurogenesis.

Cxcr4 is conserved in most vertebrates. Both *Ec_*Cxcr4a and *Ec_*Cxcr4b possess conserved domains in chemokine receptors, containing an extracellular N-terminus, seven TMs, four ECLs, four ICLs, and an intracellular C-terminus (Figure 1). Previous studies have proposed a two-step/two-site binding model and demonstrated key elements for the human SDF-1-CXCR4 interaction [48,49,93,94,95]. SDF-1 binds the CXCR4 N-terminus, and subsequently interacts with the TM region to trigger receptor activation. Using *Ec_*Cxcr4a/b chimeras and mutants (Figure 7), we determined that the N-terminus is crucial for the membrane localization and ligand binding of *Ec_*Cxcr4a/b. The best-characterized teleost chemokine ligand/receptor is zebrafish Cxcl12-Cxcr4 [4]. The zebrafish Cxcl12a–Cxcr4b and Cxcl12b–Cxcr4a interactions have been proved and the aa N33 of zebrafish Cxcl12a is responsible for the specific recognition of zebrafish Cxcr4b [96]. In this study, we confirmed the stronger interactions of *Ec_*Cxcl12a–*Ec_*Cxcr4b and *Ec_*Cxcl12b–*Ec_*Cxcr4a (Figure 6). Moreover, we found that the N-terminus contributed to the specific recognition of *Ec_*Cxcr4a-*Ec_*Cxcl12b and *Ec_*Cxcr4b-*Ec_*Cxcl12a. TM3 forms a disulfide bond with ECL2, which stabilizes the seven-trans-membrane helix bundle of chemokine receptors [97], and has been proposed to interact with G-proteins [39]. CXCR4 forms dimers for proper physiological activity [98] and TM5 is supposed to maintain the dimer motif [99]. In this study, we demonstrated that TM5 is needed for specific recognition and TM3 is not involved in the Cxcr4-Cxcl12 interaction.

In conclusion, current studies represent an example of sub-functionalization of two teleost paralogs after Ts3R, which in this case is based on biased and divergent expression patterns of *Ec_cxcr4a* and *Ec_cxcr4b*, and specific ligand–receptor recognition of *Ec_*Cxcl12a–*Ec_*Cxcr4b and *Ec_*Cxcl12b–*Ec_*Cxcr4a.

## 4. Materials and Methods

### 4.1. Experimental Fish

Adult orange-spotted groupers were bought from markets in Wuhan, China. The samples of orange-spotted groupers embryos and juveniles were obtained from the Marine Fisheries Development Center of Guangdong Province, China. All experiments and animal treatments were approved by the Institute of Hydrobiology Institutional Animal Care and Use Committee (Approval ID: keshuizhuan 0829, 2012).

### 4.2. RNA Extraction and qPCR

Total RNAs were extracted from different types of tissues, including heart, liver, spleen, pituitary, kidney, telencephalon and gonads using an SV Total RNA Isolation System (Promaga Z3100, Madison, WI, USA) according to the manufacturer’s protocol. In addition, 50 embryos (n = 3) at different development stages, including 2 hpf (32-cell stage), 9 hpf (gastrula stage), 11 hpf (neurula stage), 13 hpf (bud stage), 15 hpf (heart and liver stage), 19 hpf (muscle differentiation), 24 hpf (hatching stage), and 36 hpf were collected to extract total RNAs. Total RNA (1 μg) was used to synthesize first-strand cDNAs in a 20 μL reaction volume following the protocol of GoScript™ Reverse Transcription System (Promega A5000, Madison, WI, USA).

qPCR was performed on a CFX96TM Real-Time PCR System (Bio-Rad) using an iTaqTM Universal SYBR Green Supermix (Bio-Rad, California, USA). The reactions, protocols, and selection of internal controls were performed as described previously [45,46]. Specificity of amplification for each reaction was analyzed by dissociation curves using CFX manager software (Bio-Rad) and sequencing. Gene-specific primers (Table S1) were designed with http://biotools.nubic.northwestern.edu/OligoCalc.html. All samples were analyzed in triplicate, and relative expression levels of target genes were calculated using the 2^−ΔΔCT^ method. SPSS software (SPSS Inc.) was used for statistical analysis. A probability (*p*) of <0.05 was considered statistically significant.

### 4.3. Sequence and Phylogenetic Analyses

Orange-spotted grouper spleen SMART cDNA library was constructed according to the SMARTer PCR cDNA Synthesis Kit User Manual (Clontech 634,923). *Ec_cxcr4a/b* and *Ec_cccl12a/b* cDNAs were amplified by 3′ and 5′ RACE. The complete cDNA sequences of *Ec_cxcr4a* and *Ec_cxcr4b* were deposited in GenBank (accession numbers MH716017 and MH716018, respectively). The sequences of *Ec_cccl12a* and *Ec_cxcl12b* were obtained as *Ec_cxcr4a* and *Ec_cxcr4b*, and deposited in GenBank (accession numbers MH716019 and MH716020, respectively). Multiple amino acid sequence alignments were performed by Clustal W. Sequence identities were calculated using DNAStar 5.0. Phylogenetic analysis was conducted using Bayesian inference (BI) in MRBAYES 3.1.2 [100]. For BI tree, four independent Markov chain Monte Carlo (MCMC) chains were simultaneously run for 700,000 generations with a sample frequency of 1000 generations. The first 25% of the trees were discarded as burn-ins and the remaining tree samples were used to generate a consensus tree. Transmembrane-domain prediction was detected with SMART (http://smart.embl-heidelberg.de/) and TMHMM (http://www.cbs.dtu.dk/services/TMHMM/). Protein secondary structure predictions were made by SWISS-MODEL (https://swissmodel.expasy.org/).

The exon–intron structure was determined by the aligning mRNA and genomic sequences of *Ec_cxcr4a* and *Ec_cxcr4b*. To compare the genomic organization of other vertebrate *cxcr4* sequences, exon–intron data were obtained using the Ensembl Genome browser (http://asia.ensembl.org/index.html). Syntenic analysis was also carried out using information extracted from the Ensembl (http://asia.ensembl.org/index.html) genome and the chromosomal regions around *cxcr4s* genes in *H. sapiens* chr2, *M. musculus* chr1, *G. gallus* chr7, *L. oculatus* LG12, *D. rerio* chr6 and chr9, *Tetraodon. nigroviridis* chr2 and chr15, and *E. coioides* chr11 and chr12.

### 4.4. Probe Synthesis and In Situ Hybridization

For antisense-probe synthesis, a T7 RNA polymerase promoter was added to the end of reverse primers and a DIG RNA Labeling Kit (Roche, Mannheim, Germany) was used according to protocol. In brief, a 558-bp cDNA fragment of *Ec_*cxcr4a and an 821-bp cDNA fragment of *Ec_*cxcr4b were amplified by specific primers (Table S1). For WISH, orange-spotted grouper embryos were collected at 11 hpf, 13 hpf, 15 hpf, and 19 hpf, fixed in 4% PFA at 4 °C overnight, and stored in 100% methanol at −20 °C. WISH was performed as previously described [101,102]. Digital images were taken on a Leica S8 APO microscope.

### 4.5. Plasmid Constructs

For subcellular localization and chemotaxis assay, the full-length ORF of *Ec_*Cxcr4a, *Ec_*Cxcr4b, *Ec_*Cxcl12a or *Ec_*Cxcl12b was sub-cloned into Hind III/Kpn I sites of pEGFP-N3 vector (BD Biosciences Clontech). The N-terminus- truncated mutations or the corresponding chimeras by replacing the N-terminus, TM3 and TM5 between *Ec_*Cxcr4a and *Ec_*Cxcr4b, were produced by overlap PCR. The mutants of *Ec_*Cxcr4a and *Ec_*Cxcr4b were also sub-cloned into the pEGFP-N3 vector. All the plasmids were verified by sequence analysis.

### 4.6. Subcellular Localization

HEK293T cells were grown overnight to 80% confluence on microscopy cover-glasses in 6-well plates, and then transfected with 1 μg corresponding plasmids. After 24 h post transfection, the transfected cells on the cover-glasses were fixed with 4% paraformaldehyde for 20 min at room temperature, washed, incubated with 0.2% Triton X-100 for 30 min, and finally stained with 4’, 6-diamidino-2-phenylindole (DAPI) (50 ng/mL) for 10 min, followed by an examination on a fluorescence microscope (Leica CTR6) according to previous reports [103]. The magnification of all images is 400×.

### 4.7. Chemotaxis Assay

HEK293T cells were seeded in 6 cm plates overnight, and transiently transfected with 1 μg corresponding expression plasmids using a FuGENE HD Transfection Reagent (Promega , Madison, WI, USA) and cultured for 24h.Transmigration assay of the HEK293T cells was done in a 24-well chemotaxis chamber (8.0 mm diameter; Corning Inc, Corning, USA). Cell density was adjusted to 1 × 106/mL with DMEM out of bovine serum (Gibco, Melbourne, Australia).

For the transmigration assay, 300 μL adjusted *Eccxcr4a/b*-overexpression cells were placed in the upper chambers, and 500 μL recombinant human SDF-1α with different concentrations (0 ng/mL, 50 ng/mL, 100 ng/mL and 200 ng/mL) in serum-free DMEM media was placed in the lower chamber. The other groups were constructed by the following setting: (1–2) 300 μL *Ec_*Cxcr4a-HEK293T or *Ec_*Cxcr4b-HEK293T in the upper and 500 μL serum-free DMEM in the lower chamber; (3–4) 300 μL *Ec_*Cxcr4a-HEK293T or *Ec_*Cxcr4b-HEK293T in the upper and 500 μL *Ec_*Cxcl12a-HEK293T in the lower chamber; and (5–6) 300 μL *Ec_*Cxcr4a-HEK293T or *Ec_*Cxcr4b-HEK293T in upper and 500 μL *Ec_*Cxcl12b-HEK293T in lower chamber. The transwell plates were kept at 37 °C in 5% CO_2_ for 24 h, the polycarbonate membrane was put out, fixed with 4% paraformaldehyde for 15 min and Crystal violet (C0121, Beyotime, Wuhan, China) staining for 10 min, then observed and counted the HEK293T cells under the fluorescence Leica microscope. The chemotaxis experiments of chimeras and mutants were also constructed as described above.

## Supplementary Materials

Supplementary Materials can be found at http://www.mdpi.com/1422-0067/19/10/2943/s1. Figure S1. Nucleotide sequence and deduced amino acid sequence of Eccxcr4a. Figure S2. Nucleotide sequence and deduced amino acid sequence of Eccxcr4b. Figure S3. Nucleotide sequence and deduced amino acid sequence of Eccxcl12a. Figure S4. Nucleotide sequence and deduced amino acid sequence of Eccxcl12b. Figure S5. Multiple amino acid sequence alignment of EcCxcl12a/b protein and other vertebrate Cxcl12 proteins. Figure S6. Phylogenetic tree of vertebrate Cxcl12. Table S1. Primers and adapters used in this study. Table S2. Protein sequences similarities among vertebrate Cxcr4s. Table S3. Protein sequences similarities among of vertebrate Cxcl12s.
