# Supplementary material for: Divergent Expression Patterns and Function of Two cxcr4 Paralogs in Hermaphroditic Epinephelus coioides
Source: Int J Mol Sci. 2018 Sep 27;19(10):2943. doi: 10.3390/ijms19102943 (PMC6213054; doi:10.3390/ijms19102943)
Supplement: Supplementary file 1 [file ijms-19-02943-s001.zip › Supplementary Files/Figure Legends.docx]

Figure S1: Nucleotide sequence and deduced amino acid sequence of *Eccxcr4a*.

Figure S2: Nucleotide sequence and deduced amino acid sequence of *Eccxcr4b*.

Figure S3: Nucleotide sequence and deduced amino acid sequence of *Eccxcl12a*.

Figure S4: Nucleotide sequence and deduced amino acid sequence of *Eccxcl12b*.

Figure S5: Multiple amino acid sequence alignment of *Ec*Cxcl12a/b protein and other vertebrate Cxcl12 proteins.

Figure S6: Phylogenetic tree of vertebrate Cxcl12.

Table S1: Primers and adapters used in this study.
