# Supplementary figures and images for: Divergent Expression Patterns and Function of Two cxcr4 Paralogs in Hermaphroditic Epinephelus coioides
Source: Int J Mol Sci. 2018 Sep 27;19(10):2943. doi: 10.3390/ijms19102943 (PMC6213054; doi:10.3390/ijms19102943)

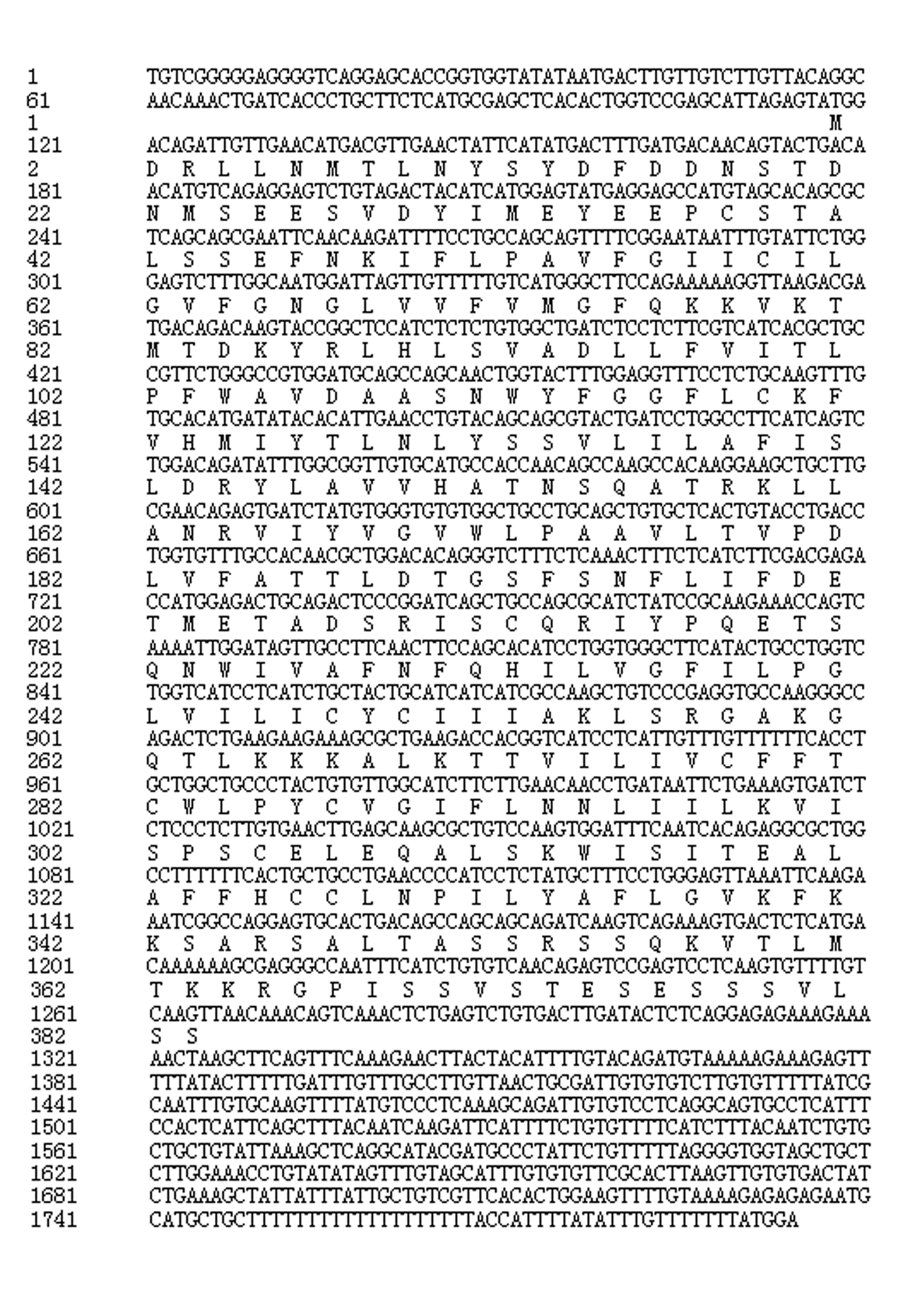

Supplement: Supplementary file 1 [file ijms-19-02943-s001.zip › Supplementary Files/Figure S1.tif]

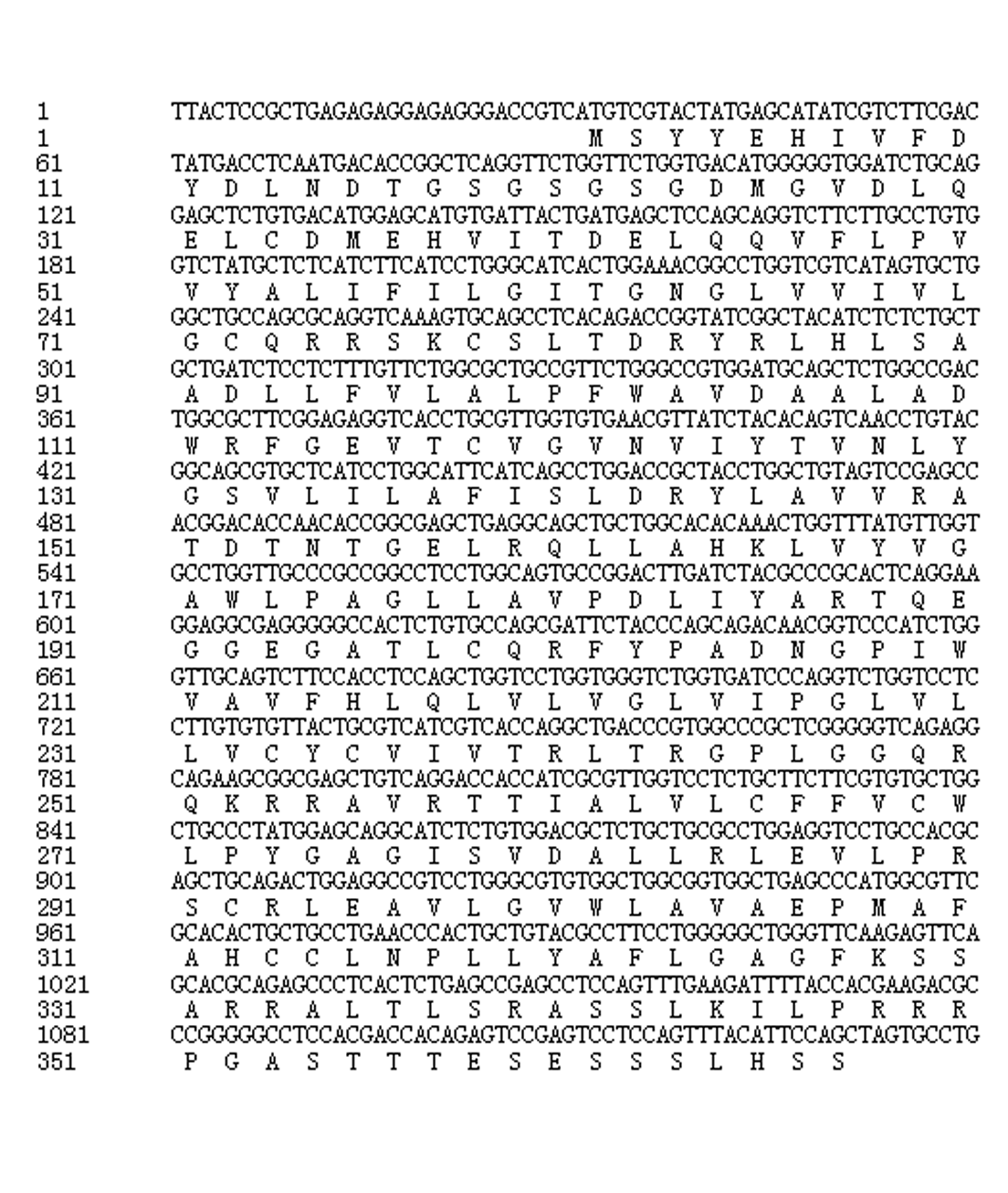

Supplement: Supplementary file 1 [file ijms-19-02943-s001.zip › Supplementary Files/Figure S2.tif]

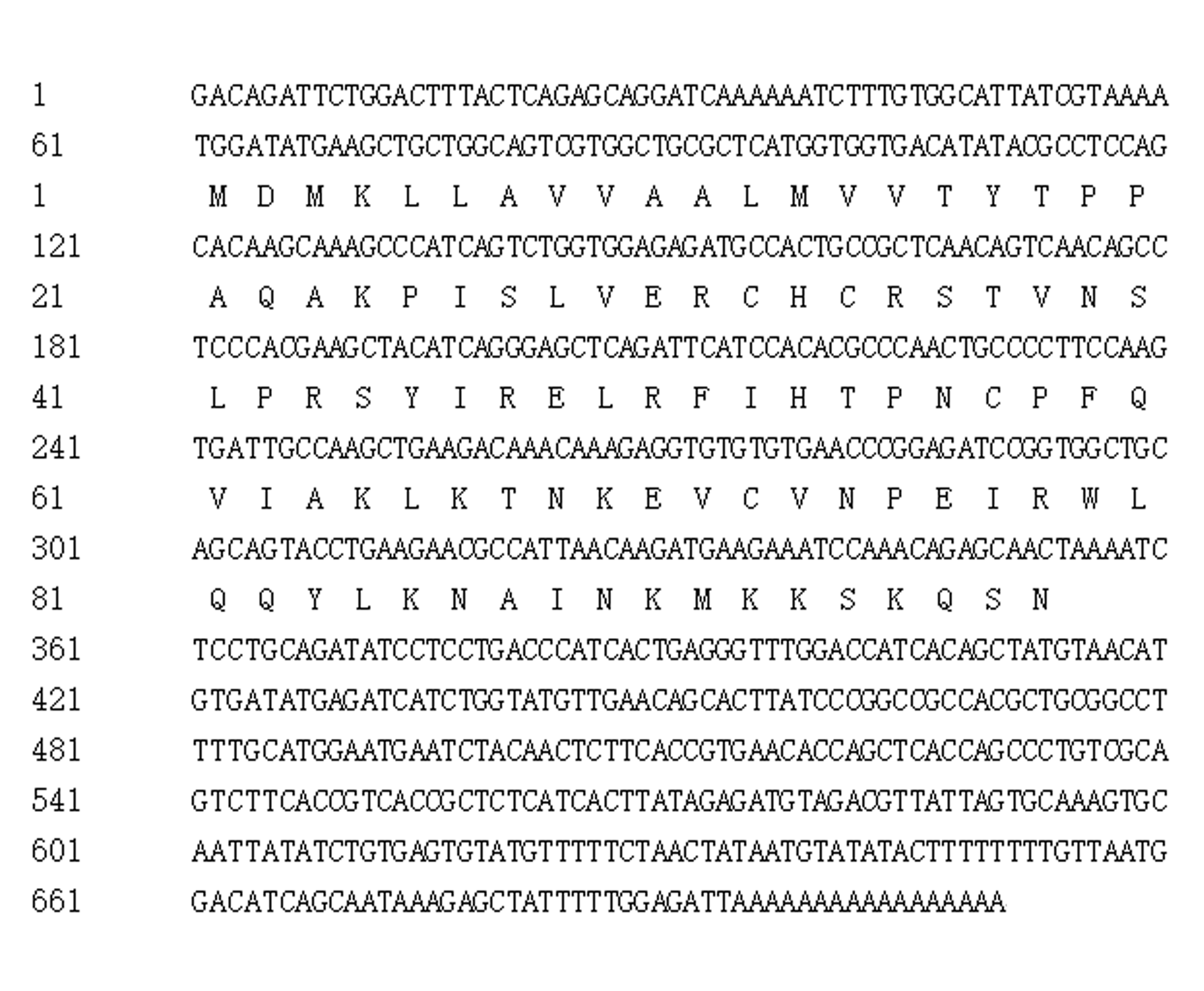

Supplement: Supplementary file 1 [file ijms-19-02943-s001.zip › Supplementary Files/Figure S3.tif]

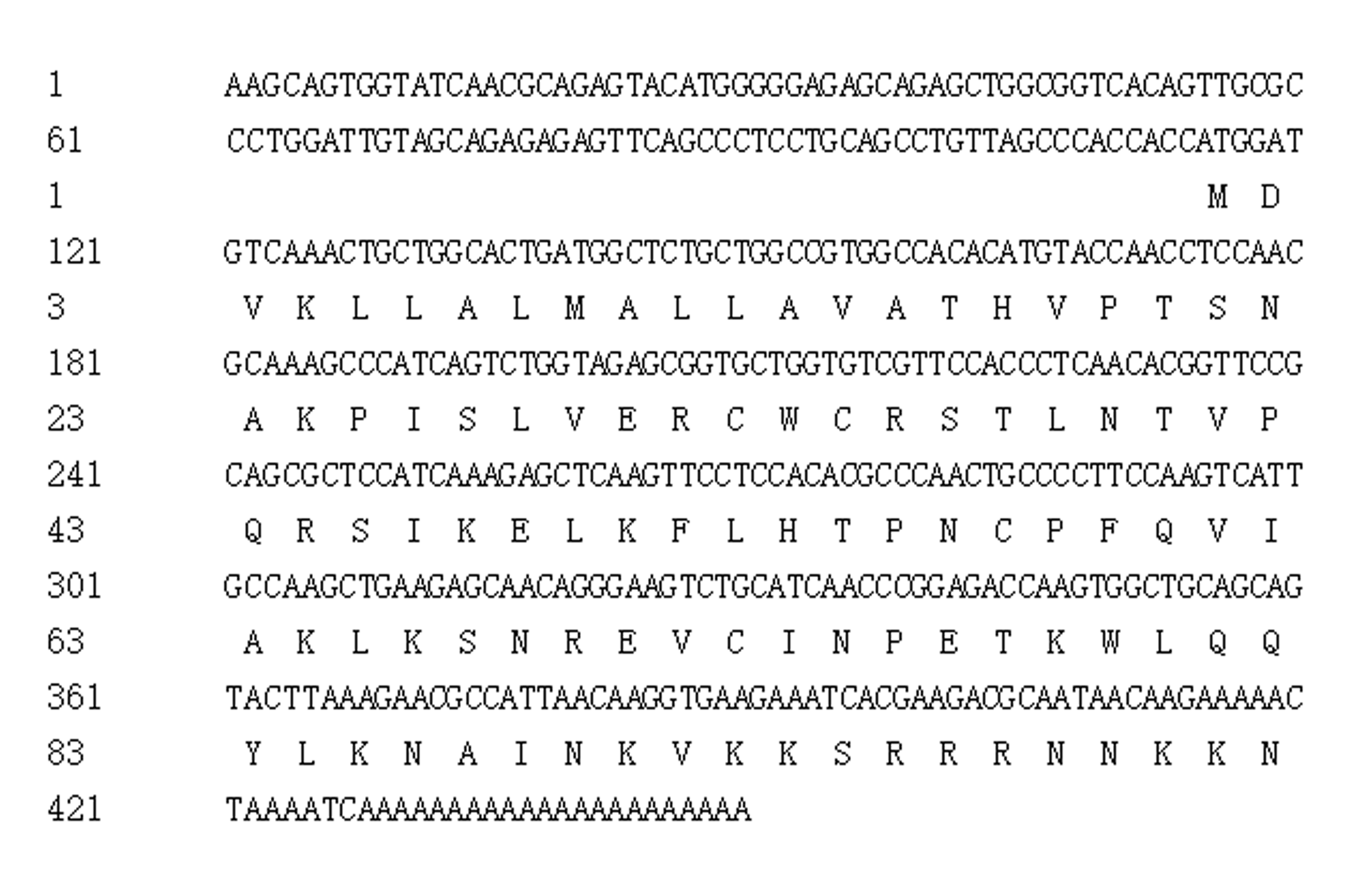

Supplement: Supplementary file 1 [file ijms-19-02943-s001.zip › Supplementary Files/Figure S4.tif]

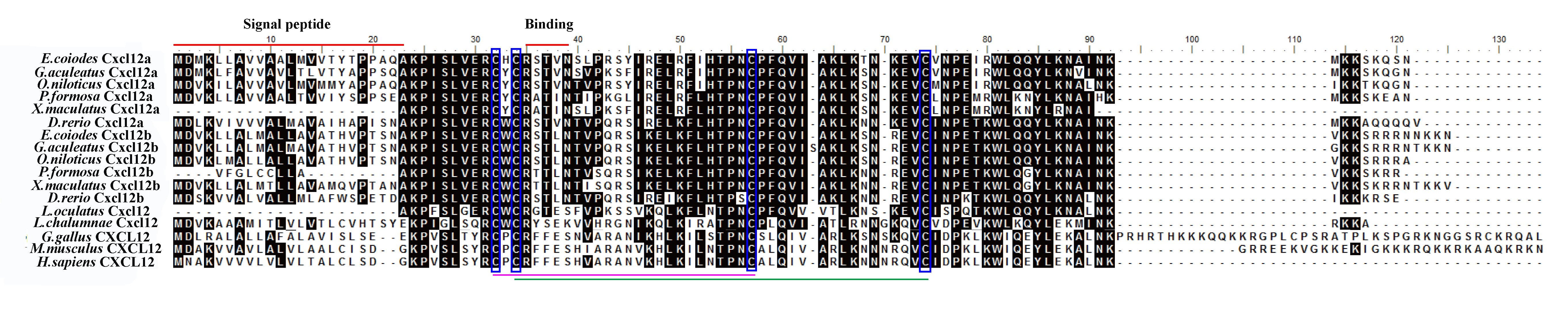

Supplement: Supplementary file 1 [file ijms-19-02943-s001.zip › Supplementary Files/Figure S5.tif]

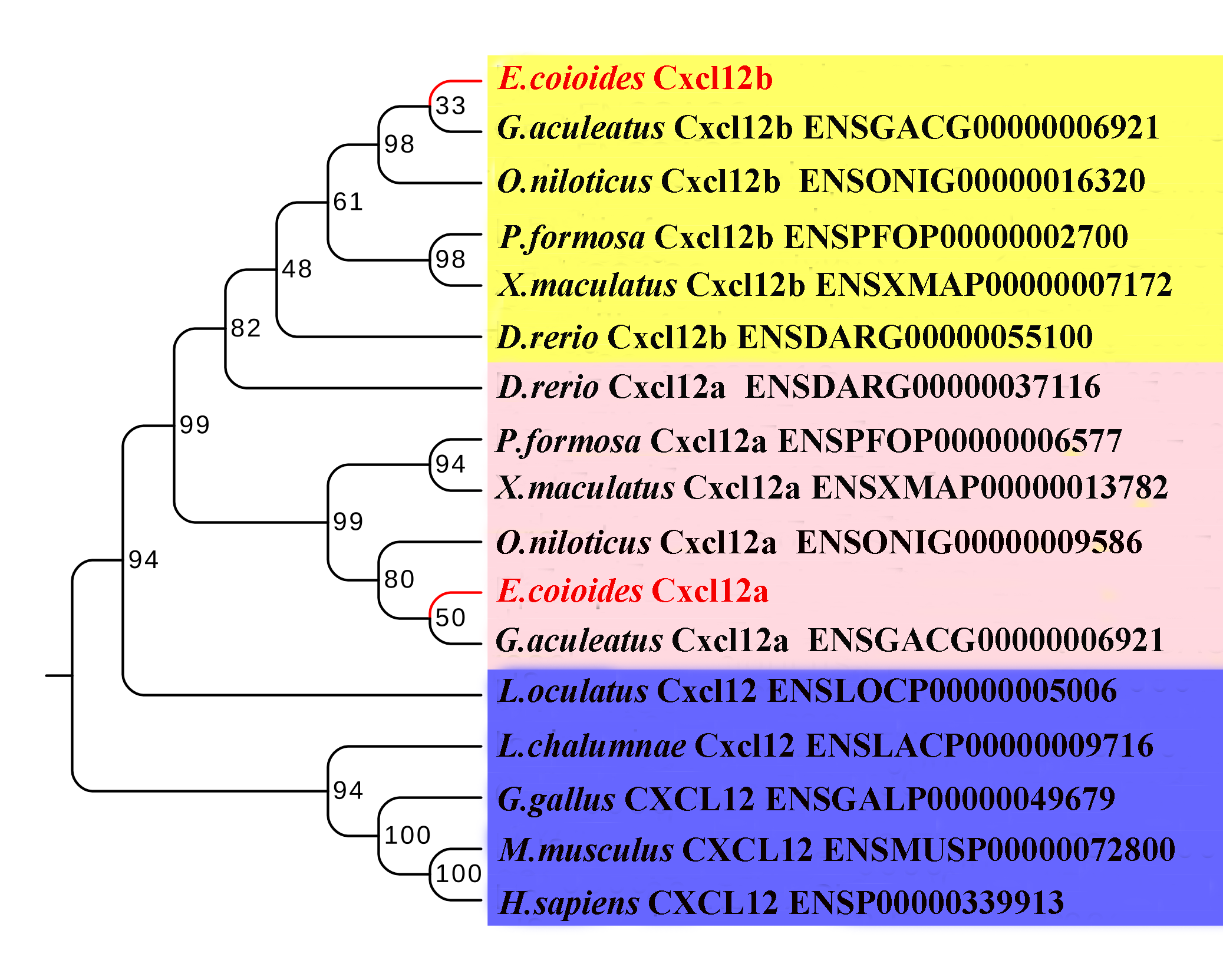

Supplement: Supplementary file 1 [file ijms-19-02943-s001.zip › Supplementary Files/Figure S6.tif]
